# Calculation of pulmonary capillary wedge pressure including left atrial function is superior to morphology alone and accurately identifies elevated filling pressures in left heart disease

**DOI:** 10.1016/j.jocmr.2025.102681

**Published:** 2025-12-28

**Authors:** Sören J. Backhaus, Ben N. Schmermund, Andreas J. Rieth, Matthias Rademann, Steffen D. Kriechbaum, Jan Sebastian Wolter, Christoph B. Wiedenroth, Alexander Schulz, Torben Lange, Julia M. Treiber, Samuel Sossalla, Andreas Schuster, Andreas Rolf

**Affiliations:** a Department of Cardiology, Campus Kerckhoff of the Justus-Liebig-University Giessen, Kerckhoff-Klinik, Bad Nauheim, Germany; b Cardio-Pulmonary Institute (CPI), Giessen/Bad Nauheim, Germany; c Department of Cardiology and Angiology, Medical Clinic I, University Hospital Giessen, Justus-Liebig-University Giessen, Giessen, Germany; d Department of Thoracic Surgery, Campus Kerckhoff of the Justus-Liebig-University Giessen, Kerckhoff-Clinic, Bad Nauheim, Germany; e University Medical Center Göttingen, Department of Cardiology and Pneumology, Georg-August University, Göttingen, Germany; f German Center for Cardiovascular Research (DZHK), Partner Site Lower Saxony, Göttingen, Germany; g Department of Medicine, Cardiovascular Division, Beth Israel Deaconess Medical Center and Harvard Medical School, Boston, Massachusetts 02215, USA; h FORUM Cardiology, Rosdorf, Germany

**Keywords:** Pulmonary hypertension, Left heart involvement, HFpEF, Atrial strain, PCWP

## Abstract

**Background:**

Right heart catheterization (RHC) with pulmonary capillary wedge pressure (PCWP) assessment is the reference standard for diagnosis of heart failure with preserved ejection fraction (HFpEF), but remains largely underused. Different approaches for non-invasive PCWP calculation have been proposed. However, as left atrial strain (LA Es) and end-systolic volume index (ESVi) emerge as key criteria in HFpEF, we sought to investigate them for PCWP calculation.

**Methods:**

The derivation population consisted of patients referred to RHC and cardiovascular magnetic resonance (CMR) imaging who were enrolled in a prospective monocentric registry. Patients were classified by RHC according to current guideline recommendations. The external validation population consisted of patients included in the HFpEF-Stress trial who underwent exercise-stress RHC and CMR with follow-up after 4 years for hospitalized cardiovascular events. Performance of strain-derived PCWP was compared to a published LA volume (LAV) and left ventricular mass (LVM) derived method.

**Results:**

The derivation population consisted of n = 209 patients, n = 123 underwent exercise-stress RHC (n = 55 without pulmonary hypertension [PH], n = 72 pre-capillary, n = 27 combined post- and pre-capillary pulmonary hypertension [CpcPH], n = 15 isolated post-capillary pulmonary hypertension [IpcPH], n = 34 exercise, and n = 6 unclassified PH). Linear regression models identified the following formulae for functional PCWPrest 10.304 − 0.095 * Es + 0.098 * ESVi and functional PCWPstress 24.666 − 0.251 * Es + 0.056 * ESVi calculation. The validation population consisted of n = 74 patients (n = 15 without, n = 5 pre-capillary, n = 8 CpcPH, n = 10 IpcPH, and n = 32 exercise PH with n = 4 remaining unclassified). Functional PCWPrest (11.8) and RHC-derived PCWPrest (11 mmHg) were statistically similar (p = 0.285) and showed moderate correlation (r = 0.53, p < 0.001). Functional PCWPrest (area under the curve [AUC] 0.80) and PCWPstress (AUC 0.85) accurately identified HFpEF patients, were superior to LAV/LVM-based PCWP (AUC 0.67, p ≤ 0.002) and showed prognostic implications (hazard ratio 1.37 (1.16–1.62) and 1.29 (1.14–1.46), p < 0.001).

**Conclusion:**

Functional PCWP may aid in the identification of post-capillary involvement in PH and HFpEF superiorly compared to morphology-derived PCWP and shows prognostic implications.

## Graphical abstract

**Figure:**
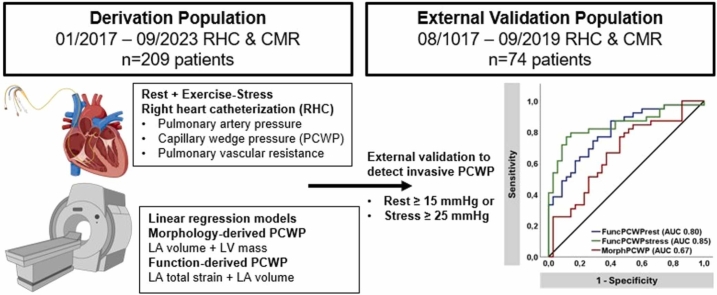

## 1 Introduction

On the one hand, pulmonary hypertension (PH) other than in left heart disease (group 2) can coincide with left heart disease, resulting in combined post- and pre-capillary pulmonary hypertension (CpcPH). On the other hand, PH can be the result of left heart disease resulting first in isolated post-capillary pulmonary hypertension (IpcPH) which can induce pulmonary vascular remodeling leading to CpcPH [1]. However, group 2 is not limited to reduced left ventricular ejection fraction (LVEF). Indeed, heart failure with preserved ejection fraction (HFpEF) is on the rise and a common reason for IpcPH and CpCPH [2]. Right heart catheterization (RHC) at rest and during exercise-stress represents the reference standard for detection and classification of PH as well as diagnosis of HFpEF by detection of elevated pulmonary capillary wedge pressure (PCWP) at rest ≥15 mmHg and during exercise-stress ≥25 mmHg [3]. Notwithstanding, RHC remains underused in patients with preserved EF and signs of diastolic dysfunction on echocardiography, potentially due to its invasive nature and limited therapeutic consequences in HFpEF. Proposed indices try to improve screening for HFpEF; however, especially intermediate scores do require further testing [4], [5].

Cardiovascular magnetic resonance (CMR) remains the reference standard for detailed cardiac morphological and functional analyses [6]. A proposed approach for non-invasive PCWP calculation based on left ventricular mass (LVM) and left atrial volume (LAV) aimed toward improved patient selection for RHC [7]. While this approach showed promising results for PCWP calculation at rest, especially for the prediction of exercise-stress–induced PCWP increase, morphology emerged inferior to left atrial (LA) function [8]. However, exercise-stress testing has become a cornerstone for early identification of masked HFpEF [3], [4], [9] or elevated filling pressures in otherwise masked left heart involvement, such as in PH [10]. Indeed, elevated filling pressures unmasked during exercise-stress only can already result in cardio-pulmonary remodeling [11] and may therefore be considered a target to slow down adverse remodeling [12]. Notwithstanding, exercise-stress CMR imaging has yet to find its way into clinical routine protocols [9]. Consequently, we aimed to assess the value of LA function at rest for prediction of both PCWP during rest and exercise-stress RHC to evaluate its gatekeeper function for referral to exercise-stress RHC in a clinical routine protocol.

## 2 Methods

### 2.1 Study population

**Fig. 1:**
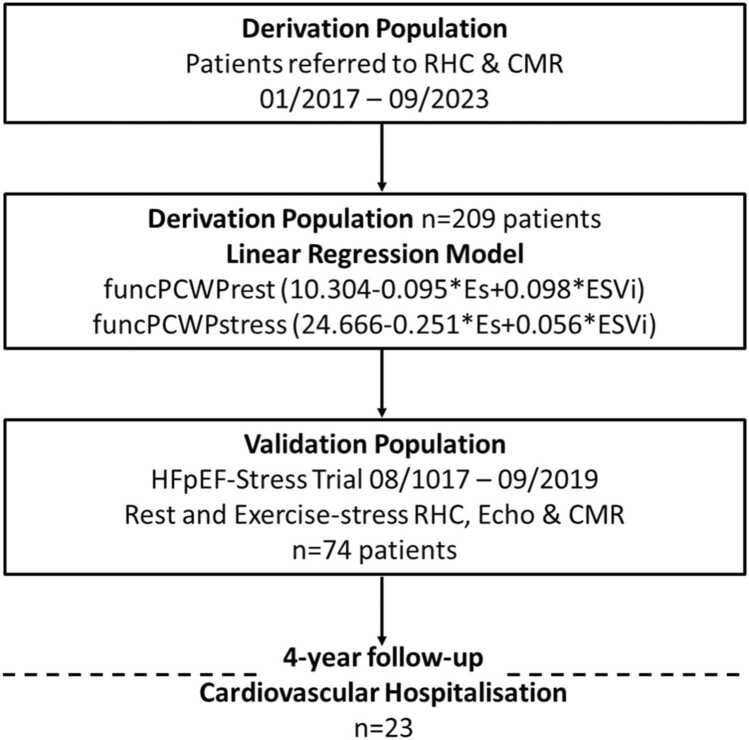
Flowchart. *CMR* cardiovascular magnetic resonance, *RHC* right heart catheterization, *PCWP* pulmonary capillary wedge pressure, *ESVi* end-systolic volume index, *HFpEF* heart failure with preserved ejection fraction

The derivation population consisted of patients referred to the imaging unit of the Kerckhoff Heart Centre who were prospectively recruited to the CMR-biobank (BioCVI) while also being referred to RHC for clinical suspicion of PH. Patients were recruited between January 2017 and June 2023 (Fig. 1). Clinical indications for CMR comprised the full spectrum of cardiovascular disease as highlighted in current guideline recommendations [13], [14]. Patients underwent CMR imaging, echocardiography, and RHC at rest. Exercise-stress RHC was included as deemed clinically necessary and feasible by the attending physician.

The external validation population consisted of the study population from the HFpEF-Stress Trial conducted at the Heart Centre Göttingen. Patients were prospectively recruited after referral for further evaluation of exertional dyspnea (New York Heart Association [NYHA] class ≥ II) in case of echocardiographic signs of diastolic dysfunction (E/e′ ≥8) and preserved LVEF ≥50%. All patients underwent rest and exercise-stress CMR, echocardiography, and RHC as well as standard laboratory testing and plethysmography for lung function evaluation. Patients were recruited between August 2017 and September 2019 [9]. The H2FPEF score was calculated for all patients [15]. A follow-up was conducted 4 years following baseline recruitment via telephone interviews and medical records for the assessment of hospitalized cardiovascular events (cardiovascular hospitalization [CVH]) [16].

The registry was approved by the local ethics committee of the University of Giessen. The HFpEF-Stress trial was approved by the local ethics committee of the University of Göttingen. Both complied with the principles of the Helsinki Declaration. All patients gave written informed consent before participation.

### 2.2 Right heart catheterization

Standard RHC was performed using a Swan-Ganz catheter introduced through a 7F sheath via the right internal jugular vein using ultrasound guidance [17]. Following strict positioning and zero adjustment of the pressure transducer, right atrial (RA), right ventricular (RV), pulmonary artery (PA), and PCWP were obtained and averaged over several respiratory cycles. Cardiac output was measured by means of thermodilution and averaged from at least 3 valid measurements. Exercise stress was conducted in supine position using bicycle ergometer stress. PH was defined according to current guideline recommendations [1]. The presence of HFpEF in the HFpEF Stress Trial was defined according to PCWP of ≥15 mmHg at rest or ≥25 mmHg during exercise stress, respectively [2], [3].

### 2.3 Echocardiography

Standard echocardiography included apical 2, 3, and 4 chamber (Ch) as well as parasternal long-axis (LAX) and short-axis (SAX) views. Doppler assessments were performed as deemed necessary, including color Doppler imaging for evaluation of valve regurgitation and continuous wave Doppler for evaluation of valve stenosis. Tricuspid annular plane systolic excursion was measured on M-Mode.

### 2.4 Cardiovascular magnetic resonance

CMR was conducted on a clinical 3.0T Magnetom Skyra MRI scanner (Siemens Healthcare, Erlangen, Germany) in a head-first supine position using commercially available array coils for cardiac imaging. Post-processing was performed using commercially available software platforms, including CVI42 (Calgary, Alberta, Canada), TomTec (2D CPA MR, Cardiac Performance Analysis, TomTec Imaging Systems, Unterschleissheim, Germany), and Medis (QMass®, Medical Imaging Systems, Leiden, Netherlands) [18].

**Fig. 2:**
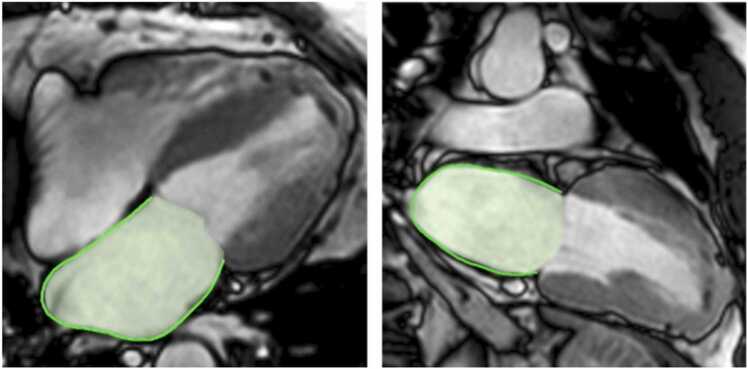
Illustration of left atrial strain and end-systolic volume assessment on a 2- and 4-chamber view of a balanced steady-state free precession cine sequence

Balanced steady-state free precession cine sequences were acquired for 2-, 3-, and 4-Ch LAX orientations as well as a SAX stack covering both ventricles. Left ventricular (LV) mass and biventricular volumetric analyses, including end-diastolic/systolic and stroke volume (EDV/ESV/SV) as well as EF, were assessed on the SAX stack. LA ESV was assessed on 2- and 4-Ch LAX views using the Simpson bi-plane approach. Deformation imaging was performed on LAX orientations as appropriate including LV global longitudinal strain (GLS) as well as left/right (LA/RA) strain (Fig. 2). Atrial strain was assessed according to reservoir function Es (venous return during ventricular systole), conduit function Ee (passive early diastolic ventricular filling), and booster pump function Ea (late diastolic ventricular filling) [19].

### 2.5 Statistical analyses

Continuous variables are given as medians with corresponding interquartile ranges (IQR). They were compared by nonparametric Mann-Whitney U testing if independent or Wilcoxon signed-rank testing if dependent. Categorical variables are reported as frequencies. They were compared using the chi-squared test.

PCWP was calculated as follows: First, a formula for morphology-derived PCWP calculations was taken from the published literature [7]:

(*morphPCWP*: 6·1352 + 0·02256 ∗ LVM + 0·07204 ∗ LAV)

Second, formulae for LA function-derived PCWP calculation based on LA Es and end-systolic volume index (ESVi) at rest were generated in the derivation cohort by means of linear regression models. Variable selection was performed based on clinical evidence, given ESVi as an established clinical morphological parameter and Es as a newly proposed addition considering it may be a more dynamic parameter. The models resulted in the following formulae for computation of

PCWP at rest (*funcPCWPrest*: 10.304 − 0.095 * Es + 0.098 * ESVi)

and

predicted PCWP for exercise-stress (*funcPCWPstress*: 24.666 − 0.251 * Es + 0.056 * ESVi).

These formulae were then tested in the validation population without any changes. Correlation was established based on Spearman rank correlation coefficient. Agreements of calculated and measured PCWP were assessed using intraclass correlation coefficient (ICC) based on two-way mixed models of absolute agreement [20]. Diagnostic accuracy was evaluated by means of sensitivity/specificity/positive and negative predictive value (PPV/NPV) as well as area under the receiver-operator characteristic (ROC) curve (AUC) analyses, which are reported with 95% confidence intervals (CI). AUC comparisons were calculated using the method proposed by DeLong et al. [21]. Prognostic implications were identified using first univariate and multivariable Cox proportional hazards models, which were based on continuous variables reporting hazard ratios (HR) with corresponding 95% CI, and second, Kaplan-Meier plots with associated log-rank testing. A two-tailed p-value <0.05 was considered statistically significant. Calculations were performed using SPSS version 29.0.2.0 (IBM, Armonk, New York) and MedCalc version 23.1.3 (MedCalc Software BVBA, Ostend, Belgium).

## 3 Results

### 3.1 Study populations

The derivation population consisted of n = 209 patients, n = 123 of whom underwent exercise-stress RHC. PH was classified as absent n = 55, pre-capillary n = 72, CpcPH n = 27, IpcPH n = 15, exercise-induced n = 34, and n = 6 patients remained unclassified. The validation population consisted of 74 patients. PH was classified as absent n = 15, pre-capillary n = 5, CpcPH n = 8, IpcPH n = 10, and exercise PH n = 32 with n = 4 remaining unclassified. Twenty-three CVH were noted within the 4-year follow-up. The median H2FPEF score was 4 (3, 5 IQR).

The derivation population was older and with more severely impacted cardiac physiology as appreciated from higher NYHA-class, NTproBNP, tricuspid annular plane systolic excursion (p < 0.001). There was no difference in the degree of mitral regurgitation (p = 0.084) or PCWP (p = 0.736) at rest. Exercise-stress testing revealed increased PCWP (p = 0.004) and CI (p < 0.001) but lower PA (p = 0.042) pressure in the validation population (Table 1).

**Table 1:** Patients characteristics.

Variable	Derivation population n = 209*	Validation population n = 74	Significance p
Age (y)	62 (50, 71)	69 (64, 73)	<0.001
Sex male/female	120/89	29/45	0.007
BMI (kg/m² BSA)	27.4 (23.6, 30.8)	27.9 (26.1, 32.5)	0.044
NYHA	I 68 II 41 III 63 IV 18	I 0 II 52 III 22 IV 0	<0.001
*Laboratory testing*			
NTproBNP (ng/L)	596 (140, 2015)	133 (69, 297)	<0.001
*Echocardiography*			
TAPSE (mm)	18 (15, 21)	23 (21, 26)	<0.001
Mitral regurgitation grade	0 85 1 102 2 9	0 38 1 35 2 1	0.285
*Right heart catheterization*			
PCWP rest (mmHg)	10 (8, 14)	11 (8, 14)	0.981
PCWP stress (mmHg)	18 (15, 25)	24 (18, 28)	0.004
PA rest (mmHg)	23 (18, 31)	19 (17, 24)	<0.001
PA stress (mmHg)	43 (36, 53)	40 (34, 47)	0.042
PVR rest (Wood Units)	2.5 (1.7, 3.8)	1.8 (1.2, 2.0)	<0.001
PVR stress (Wood Units)	2.6 (1.8, 4.3)	1.5 (1.2, 2.1)	<0.001
Cardiac index rest (L/m² BSA)	2.4 (1.9, 2.8)	2.9 (2.6, 3.2)	<0.001
Cardiac index stress (L/m² BSA)	3.9 (3.0, 5.2)	5.3 (4.3, 6.4)	<0.001

*BMI* body mass index, *NYHA* New York Heart Association class, *TAPSE* tricuspid annular plane systolic excursion, *PCWP* pulmonary capillary wedge pressure, *PA* pulmonary artery pressure, *PVR* pulmonary vascular resistance, *BSA* body surface area, *NTproBNP* N-terminal pro-B-type brain natriuretic pept

* Stress right heart catheterization (n = 127)

CMR-derived volumetric and functional analyses are reported in Table 2. Overall, cardiac function was superior in the validation population. *FuncPCWPrest* (p = 0.368) and *funcPCWPstress* (p = 0.168) were similar between both populations, while *morphPCWP* was lower in the derivation population (p = 0.005).

**Table 2:** Cardiovascular magnetic resonance imaging at rest.

Variable	Derivation population n = 209	Validation population n = 74	Significance p
*Left ventricle*			
LV mass (g/m² BSA)	56.0 (47.3, 64.6)	58.2 (50.9, 70.1)	0.046
LV EDV (mL/m² BSA)	76.3 (64.9, 91.8)	69.3 (59.3, 78.2)	0.003
LV ESV (mL/m² BSA)	32.2 (26.9, 41.3)	20.4 (15.0, 26.5)	<0.001
LV SV (mL/m² BSA)	42.4 (35.1, 49.1)	49.6 (42.0, 54.9)	<0.001
LVEF (%)	57.0 (50.8, 62.8)	68.5 (65.2, 75.9)	<0.001
FT LV GLS (%)	−15.2 (−12.0, −18.0)	−17.4 (−15.6, −19.0)	<0.001
*Left atrium*			
FT LA Es (%)	26.7 (13.8, 34.7)	30.1 (22.9, 37.7)	0.009
FT LA Ee (%)	10.7 (5.6, 16.4)	13.4 (9.4, 19.0)	0.001
FT LA Ea (%)	13.7 (6.8, 19.0)	15.5 (9.5, 19.6)	0.277
LAVI (mL/m² BSA)	35.0 (24.7, 51.3)	45.1 (32.3, 54.1)	0.004
Right ventricle*			
RV EDV (mL/m² BSA)	88.7 (72.4, 110.9)	67.1 (57.1, 74.7)	<0.001
RV ESV (mL/m² BSA)	45.1 (34.0, 64.1)	21.6 (18.5, 28.0)	<0.001
RV SV (mL/m² BSA)	41.5 (33.8, 49.2)	43.8 (37.9, 50.9)	0.092
RV EF (%)	48.2 (39.1, 55.0)	65.0 (61.0, 70.4)	<0.001
*Right atrium*			
FT RA Es (%)	22.3 (13.2, 29.4)	43.9 (32.3, 49.9)	<0.001
FT RA Ee (%)	10.1 (5.0, 14.7)	23.0 (15.7, 29.5)	<0.001
FT RA Ea (%)	10.9 (6.1, 15.8)	18.2 (13.2, 23.3)	<0.001
*CMR-derived PCWP*			
CalcPCWPrest	11.2 (9.7, 13.7)	11.8 (10.3, 13.5)	0.368
CalcPCWPstress	19.8 (17.6, 24.2)	19.5 (17.0, 21.7)	0.168
CalcPCWP LAV	13.9 (12.1, 15.8)	15.2 (12.9, 16.8)	0.005

*LV* left ventricular, *EDV/ESV* end-diastolic/-systolic volume, *SV* stroke volume, *EF* ejection fraction, *FT* feature-tracking, *GLS* global longitudinal strain, *LA* left atrial, *Es/Ee/Ea* atrial reservoir/conduit/booster pump function, *LAVI* left atrial volume index, *RV* right ventricular, *RA* right atrial, *Calc* calculated, *PCPW* pulmonary capillary wedge pressure

* RV n = 208

### 3.2 Agreement of PCWP

R² was 0.26 for both linear regression models, *funcPCWPrest*/RHC-derived PCWPrest and *funcPCWPstress*/RHC-derived PCWPstress. *FuncPCWPrest* and RHC-derived PCWPrest did not show statistically significant differences (11.8 vs 11.0 mmHg, p = 0.285), while *morphPCWP* was higher compared to RHC-derived PCWPrest (15.2 vs 11.0 mmHg, p < 0.001). *FuncPCWPstress* was lower than RHC-derived PCWPstress (19.5 vs 24.0 mmHg, p < 0.001). *FuncPCWPrest* and RHC-derived PCWPrest (r = 0.53, p < 0.001) as well as *funcPCWPstress* and RHC-derived PCWPstress (r = 0.62, p < 0.001) showed moderate correlation, while *morphPCWP* and RHC-derived PCWPrest correlated less accurately (r = 0.38, p = 0.001). Similarly, ICC were fair for funcPCWPrest/RHC-derived PCWPrest (ICC = 0.59) and *funcPCWPstress*/RHC-derived PCWPstress (ICC = 0.53), while being lower and fair for *morphPCWP*/RHC-derived PCWPrest (ICC = 0.45) and poor for *morphPCWP*/RHC-derived PCWPstress (ICC = 0.27).

### 3.3 Functional PCWP—diagnostic accuracy

**Fig. 3:**
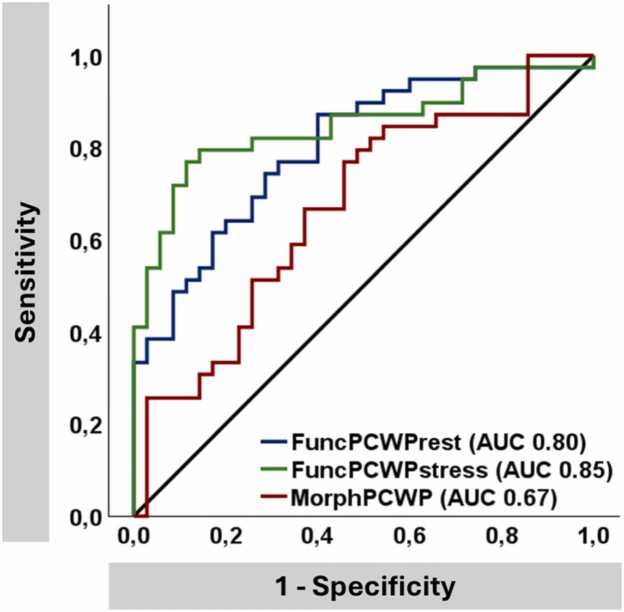
AUC analyses. The figure displays the diagnostic accuracy to detect patients with increased pulmonary capillary wedge pressure (PCWP) ≥15 mmHg at rest and/or ≥25 mmHg during exercise-stress as area under the curve (AUC)

*FuncPCWPrest* and *funcPCWPstress* accurately identified patients with either PCWPrest ≥15 mmHg or PCWPstress ≥25 mmHg (AUC 0.80 vs 0.85, p = 0.107) and were superior to *morphPCWP* (AUC 0.67, p < 0.001 and p = 0.002), Fig. 3. *FuncPCWPstress* (p = 0.039) but not *funcPCWPrest* (p = 0.298) was superior to the H2FPEF score (AUC 0.74) in identifying HFpEF patients. Among all patients with exercise PH, *funcPCWPrest* and *funcPCWPstress* accurately identified HFpEF patients (AUC 0.77 and 0.76).

Dichotomization at the Youden index revealed better diagnostic accuracy of *FuncPCWPrest* 10.9 mmHg (sensitivity 87%, specificity 60%, PPV 71%, NPV 81%) and *funcPCWPstress* 19.7 mmHg (sensitivity 77%, specificity 89%, PPV 88%, NPV 78%) as opposed to *morphPCWP* 14.3 mmHg (sensitivity 77%, specificity 54%, PPV 65%, NPV 68%).

### 3.4 Functional PCWP—prognostic significance

**Fig. 4:**
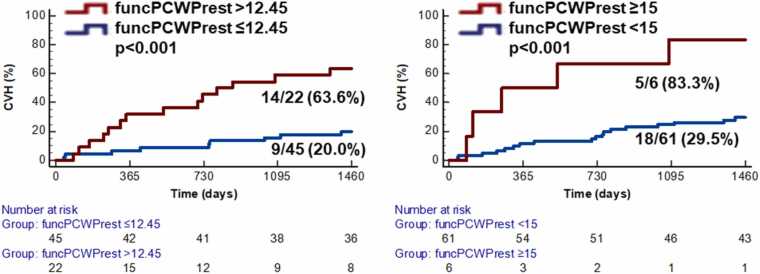
Kaplan-Meier plot—cardiovascular hospitalization (CVH). The graphs show the percentage of patients who were hospitalized due to cardiovascular reasons according to functional resting PCWP (funcPCWPrest) dichotomized at the Youden index (left) and established cut-off of 15. *PCWP* pulmonary capillary wedge pressure

*FuncPCWPrest* (HR 1.37, 95% CI 1.16–1.62, Wald 13.37, p < 0.001) and *funcPCWPstress* (HR 1.29, 95% CI 1.14–1.46, Wald 16.78, p < 0.001) were significantly associated with CVH. This finding was independent of LV GLS and RV EF (HR 1.39, 95% CI 1.14–1.70, Wald 10.29, p < 0.001 and HR 1.36, 95% CI 1.15–1.60, Wald 13.12, p < 0.001). After dichotomization of *funcPCWPrest* identified by the Youden index (12.45 mmHg) as well as the commonly established cut-off of 15 mmHg for invasive PCWP at rest, Kaplan-Meier plots confirmed prognostic significance (p < 0.001 for both, Fig. 4).

## 4 Discussion

Based on prospectively recruited derivation and validation PH populations, the present study elaborates on PCWP calculation based on atrial functional assessments. First, *funcPCWPrest* emerges similar to RHC-derived PCWPrest, while predicted *funcPCWPstress* as appreciated from atrial function at rest underestimates RHC-derived PCWPstress. Second, *funcPCWPrest/stress* accurately identifies post-capillary involvement in PH and emerges superior to in the literature published approaches including LAV and LVM (*morphPCWP*). Lastly, *funcPCWPrest*/*stress* bears prognostic implications for CVH.

There was no statistically significant difference between *funcPCWPrest* and RHC-derived PCWPrest. This finding is of particular interest. First, because both the derivation and validation populations showed differences in terms of PH subtype and subsequently overall cardiac volumetric and functional analyses. Second, because strain assessments have been performed using different commercially available software solutions. Indeed, intervendor variability of strain assessments has previously been discussed with focus on ventricular deformation imaging [18], [22]. The finding of accurate agreement of *funcPCWPrest* and RHC-derived PCWP thus underlines overall improved reproducibility of atrial strain as well as applicability of the present approach for PCWP calculation. This may promote clinical implementation. Indeed, the linear regression model could enable PCWP calculation following LA strain assessment as readily available from FT post-processing software. However, automated CMR-derived PCWP calculation would require further dedicated prospective validation.

Previously developed models for PCWP calculation based on LAV [7] or area [23] and LVM demonstrated good diagnostic and prognostic value [24]. However, it has been shown that PCWP calculation based on morphology (atrial size and LVM) underestimates RHC-derived PCWP, especially during exercise stress, even if atrial volumes are assessed during exercise-stress as well [8]. Potentially, this may arise from atrial size not responding dynamically to exertion thus not accurately reflecting live hemodynamic changes. LA function has shown incremental value to LA size alone for the detection of HFpEF [9], [25]. Although—based on atrial function at rest—predicted *funcPCWPstress* underestimated RHC-derived PCWPstress, correlation of both approaches was high and even numerically higher during exercise stress compared to resting conditions. Both *funcPCWPrest* (AUC 0.80) and *funcPCWPstress* (AUC 0.85) showed high diagnostic accuracy to identify HFpEF patients as defined by rest and exercise-stress RHC. Both models based on atrial function outperformed the model based on morphology alone. As a result, atrial function may be considered superior for the estimation of PCWP during exercise stress. Exercise-stress CMR has not been performed in the derivation population, which is likely the case for most clinical CMR studies. LA longitudinal deformation during exercise-stress CMR has previously been shown to have the highest diagnostic accuracy to detect HFpEF as defined by rest and exercise-stress RHC-derived PCWP [9]. Consequently, LA function-derived PCWP may overcome the challenge of underestimating the dynamic range of PCWP during exercise stress. The present findings may spark future interests in PCWP calculation based on atrial function during exercise stress.

Intriguingly, although not reaching statistical significance compared to *funcPCWPrest*, *funcPCWPstress* showed the highest diagnostic accuracy for the detection of HFpEF. Importantly, exercise PH has recently been introduced to the guidelines [1] and at rest about 1/3 of all HFpEF patients are overlooked (masked HFpEF) [3]. *FuncPCWPrest/stress* accurately identified HFpEF patients among the exercise-PH group with only mild reduction in AUC (0.77 and 0.76) compared to the overall PH population. This further highlights the value of atrial functional assessments in the detection of early HFpEF. Clinical scores have been introduced for non-invasive identification of HFpEF [15]. Notwithstanding, a lot of patients are represented in the midrange of these scores and not classified as HFpEF or non-cardiac dyspnea with diagnostic certainty. Especially masked HFpEF may remain undiagnosed. Interestingly, *funcPCWPstress* but not *funcPCWPrest* outperformed the clinical H2FPEF score on AUC comparison, highlighting the additional clinical benefit [15].

PCWP and its disproportionate increase during exertion are linked to symptom severity and outcome [26], [27]. PCWP based on atrial size bears prognostic implications similar even superior to RHC-derived PCWP at rest [7]. These findings are in line with the present results. Indeed, both *funcPCWPrest/stress* showed prognostic implications, which were independent of LV GLS and RV EF. As LV GLS had previously demonstrated incremental prognostic value in HFpEF [28] and with otherwise limited prognostic information, this finding shows a promising clinical impact.

## 5 Limitations

The derivation and validation populations were not matched in terms of patients’ baseline characteristics and frequency of PH groups based on invasive hemodynamics. This introduces the general limitations of studies without prospective and random but matched allocation based on prespecified patient characteristics. However, the derivation and validation populations were assigned from prospectively recruited patient populations from two different tertiary cardiology centers employing different post-processing software. This setting may rather represent the real-world scenario where different centers may see different patient populations or employ a range of software solutions from different vendors which also introduces variability from intervendor agreements in FT [20]. Notwithstanding, similarities of *funcPCWPrest* and RHC-derived PCWP in this setting highlight potential future clinical applicability. Sample sizes of PH subtypes were small, limiting statistical analyses and power for subgroup analyses. The present linear approach for PCWP calculation was derived from a published model but exchanging LV mass for LA function based on physiological considerations. This meant to evaluate the value of dynamic LA function as opposed to constant mass. Therefore, no statistical steps were taken for considerations of multiple testing, which may result in p-value inflation or overfitting of the model. Furthermore, limitations of a sole linear model compared to potentially very dynamic invasive PCWP need to be considered. Therefore, the presented results may be considered hypothesis-generating rather than confirmatory.

## 6 Conclusion

Atrial function-derived PCWP more accurately identifies elevated filling pressures as appreciated from increased PCWP in HFpEF patients and post-capillary involvement in PH compared to morphology-derived PCWP alone. Furthermore, calculated PCWP indicates prognostic implications. It may thus aid in the detection of LV involvement in PH and diastolic dysfunction in patients with otherwise unexplained exertional dyspnea or decision-making on referral for dedicated exercise-stress RHC.

## Funding

The Kerckhoff Biomarker Registry (Bioreg) is funded by the Kerckhoff Heart Research Institute (KHFI) and the German Center for Cardiovascular Research (DZHK).

## Author contributions

**Alexander Schulz:** Writing – review & editing, Formal analysis, Data curation. **Torben Lange:** Writing – review & editing, Data curation. **Julia M. Treiber:** Writing – review & editing, Data curation. **Samuel Sossalla:** Writing – review & editing, Software, Resources, Project administration. **Jan Sebastian Wolter:** Writing – review & editing, Data curation. **Christoph B. Wiedenroth:** Writing – review & editing, Methodology, Data curation. **Steffen D. Kriechbaum:** Writing – review & editing, Methodology, Data curation. **Sören J. Backhaus:** Writing – review & editing, Writing – original draft, Visualization, Validation, Supervision, Software, Resources, Project administration, Methodology, Investigation, Formal analysis, Data curation, Conceptualization. **Andreas Schuster:** Writing – review & editing, Supervision, Software, Resources, Project administration, Methodology, Funding acquisition, Conceptualization. **Ben N. Schmermund:** Formal analysis, Data curation. **Andreas Rolf:** Writing – review & editing, Supervision, Software, Resources, Project administration, Methodology, Funding acquisition, Data curation, Conceptualization. **Andreas J. Rieth:** Writing – review & editing, Methodology, Data curation. **Matthias Rademann:** Writing – review & editing, Methodology, Data curation.

## Ethics approval and consent

The study was approved by the Ethics Committee of the University Hospital Giessen and Göttingen and complied with the Declaration of Helsinki. All individuals gave written informed consent before participating in the study.

## Consent for publication

Not applicable.

## Declaration of competing interests

The authors declare that they have no known competing financial interests or personal relationships that could have appeared to influence the work reported in this paper.

## Data Availability

Regarding data availability, we confirm that all relevant data are within the paper and all data underlying the findings are fully available without restriction and can be accessed at the Kerckhoff Heart Research Institute (KHFI) by researchers who meet the criteria for access to confidential data.
