# Supplementary material for: Calculation of pulmonary capillary wedge pressure including left atrial function is superior to morphology alone and accurately identifies elevated filling pressures in left heart disease
Source: J Cardiovasc Magn Reson. 2025 Dec 28;28(1):102681. doi: 10.1016/j.jocmr.2025.102681 (PMC13126477; doi:10.1016/j.jocmr.2025.102681)
Supplement: Supplementary file 1 — Supplementary material [file mmc1.docx]

**Supplements**


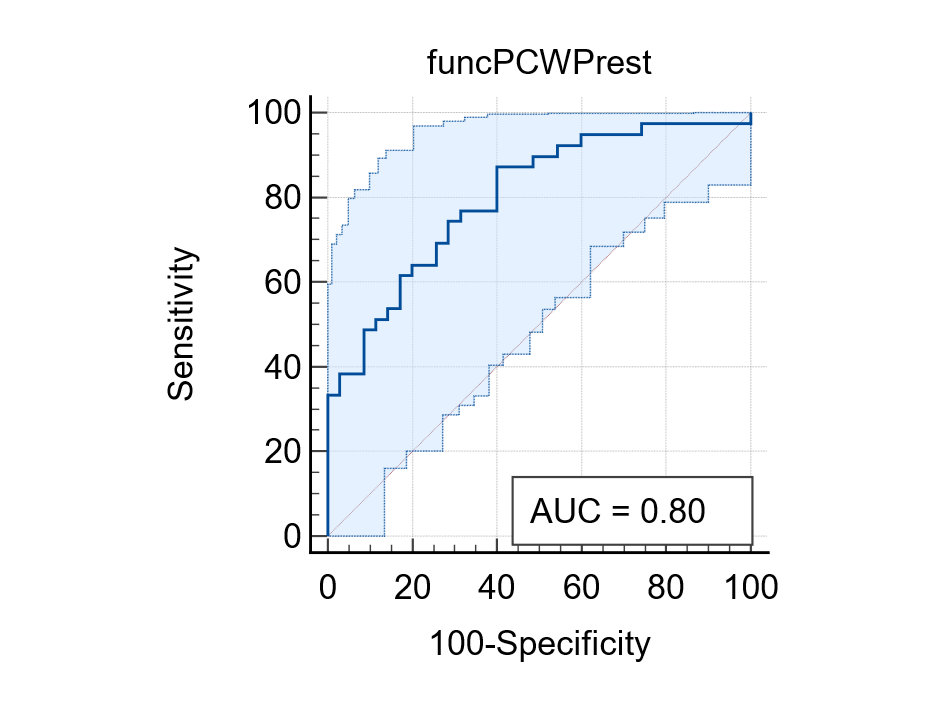


**Figure S1 AUC analyses**

The figure displays the diagnostic accuracy of funcPCWPrest to detect patients with increased pulmonary capillary wedge pressure (PCWP) ≥15 mmHg at rest and/or ≥25 mmHg during exercise-stress as area under the curve (AUC).


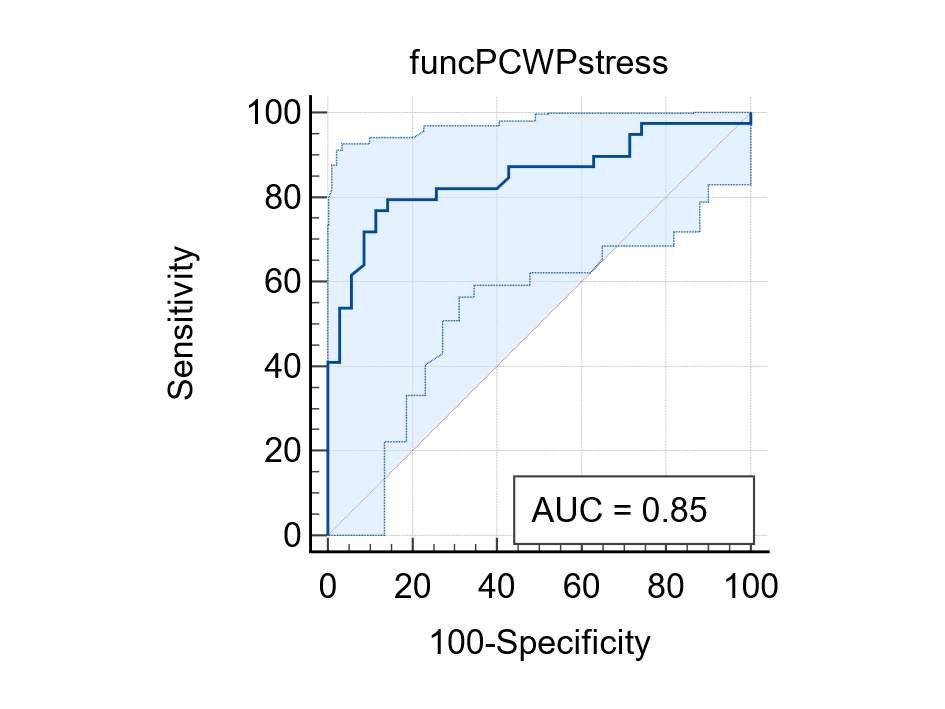


**Figure S2 AUC analyses**

The figure displays the diagnostic accuracy of funcPCWPstress to detect patients with increased pulmonary capillary wedge pressure (PCWP) ≥15 mmHg at rest and/or ≥25 mmHg during exercise-stress as area under the curve (AUC).


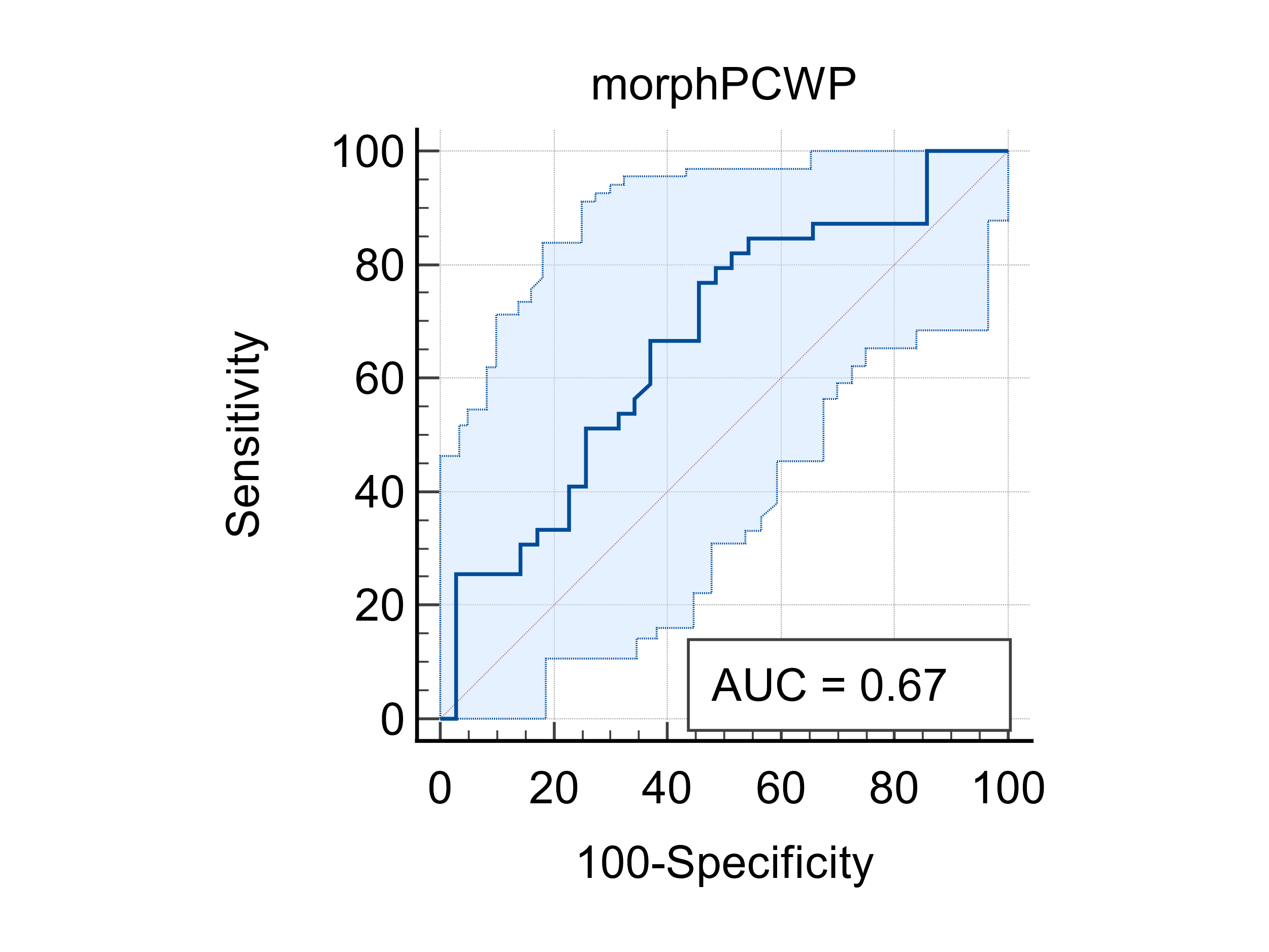


**Figure S3 AUC analyses**

The figure displays the diagnostic accuracy of morphPCWP to detect patients with increased pulmonary capillary wedge pressure (PCWP) ≥15 mmHg at rest and/or ≥25 mmHg during exercise-stress as area under the curve (AUC).
